# Enhanced Photocatalytic Activity of Spherical Nd^3+^ Substituted ZnFe_2_O_4_ Nanoparticles

**DOI:** 10.3390/ma14082054

**Published:** 2021-04-19

**Authors:** Loan T. T. Nguyen, Hang T. T. Nguyen, Thieng H. Le, Lan T. H. Nguyen, Hai Q. Nguyen, Thanh T. H. Pham, Nguyen D. Bui, Ngan T. K. Tran, Duyen Thi Cam Nguyen, Tan Van Lam, Thuan Van Tran

**Affiliations:** 1 Faculty of Chemistry, Thai Nguyen University of Education, Thai Nguyen 24000, Vietnam; lehuuthieng@dhsptn.edu.vn (T.H.L.); lannth.chem@tnue.edu.vn (L.T.H.N.); hainq@tnue.edu.vn (H.Q.N.); thanhpth@tnue.edu.vn (T.T.H.P.); ducnguyen@tnue.edu.vn (N.D.B.); 2 Faculty of Fundamental Sciences, Thai Nguyen University of Technology, Thai Nguyen 24000, Vietnam; thuyhangtnut@gmail.com; 3 NTT Hi-Tech Institute, Nguyen Tat Thanh University, Ho Chi Minh 700000, Vietnam; ttkngan@ntt.edu.vn (N.T.K.T.); ntcduyen@ntt.edu.vn (D.T.C.N.); 4 Center of Excellence for Green Energy and Environmental Nanomaterials, Nguyen Tat Thanh University, Ho Chi Minh 700000, Vietnam

**Keywords:** zinc ferrite, neodymium-doping, photo-Fenton, Rhodamine B

## Abstract

In this study, nanocrystalline ZnNd_x_Fe_2−x_O_4_ ferrites with x = 0.0, 0.01, 0.03 and 0.05 were fabricated and used as a catalyst for dye removal potential. The effect of Nd^3+^ ions substitution on the structural, optical and photo-Fenton activity of ZnNd_x_Fe_2−x_O_4_ has been investigated. The addition of Nd^3+^ ions caused a decrease in the grain size of ferrites, the reduction of the optical bandgap energies and thus could be well exploited for the catalytic study. The photocatalytic activity of the ferrite samples was evaluated by the degradation of Rhodamine B (RhB) in the presence of H_2_O_2_ under visible light radiation. The results indicated that the ZnNd_x_Fe_2−x_O_4_ samples exhibited higher removal efficiencies than the pure ZnFe_2_O_4_ ferrites. The highest degradation efficiency was 98.00%, attained after 210 min using the ZnNd_0.03_Fe_1.97_O_4_ sample. The enhanced photocatalytic activity of the ZnFe_2_O_4_ doped with Nd^3+^ is explained due to the efficient separation mechanism of photoinduced electron and holes. The effect of various factors (H_2_O_2_ oxidant concentration and catalyst loading) on the degradation of RhB dye was clarified.

## 1. Introduction

It has been proved that the discharge of organic compounds, including dyes, from manufacturing plants led to growing contamination in the aquatic ecosystem [1,2]. There are numerous impacts of color pollution, so more and more techniques have been found to address this environmental problem [3]. Among them, photocatalysis, which relies on semiconductors and irradiation-based degradation of organic substances, is an effective method [4,5]. The benefits of this approach include environmental friendliness, the potential to entirely decompose organic pollutants into inorganic molecules, i.e., CO_2_ and H_2_O. TiO_2_ [6,7,8], WO_3_ [9,10,11,12,13], and BiVO_4_ [14,15] are typical photocatalysts that have been well studied for dyes degradation. Nano ferrites, another material, have recently received a great deal of interest due to their high stability, strong magnetic properties, and high catalytic performance. In particular, such nanoparticles may be used as a photocatalyst under visible light in wastewater treatment thanks to their narrow band gap [16,17]. In addition, the method used for ferrite synthesis may differ depending on the desired characteristics, and a number of synthesis routes have been investigated so far, like solvothermal [18], sol-gel [19], coprecipitation [20], and combustion method [21]. For example, Xiaojun Guo et al. [17] reported that the NiFe_2_O_4_ hollow nanospheres synthesized by solvothermal method had a high photoactivity for methylene blue (MB) degradation, which achieved the removal efficiency of approximately 98.5% only within 50 min in the presence of 5 mM H_2_O_2_ and 0.06 g·L^−1^ H_2_C_2_O_4_. In other extensive studies on ferrite, ZnFe_2_O_4_ nanomaterial has been documented to be effective in removing a wide range of organic compounds, such as Orange II [22], Red 88, Acid Orange 8, Malachite Green [23], Congo red [24], methylene blue [25] and tetracycline [26]. Besides, MnFe_2_O_4_, CoFe_2_O_4_, CuFe_2_O_4_ and MgFe_2_O_4_ nanospinels have been reported to show effective photocatalytic activity to eliminate distinct categories of dyes [19,20,27,28]. Because of the fact that ferrite catalysts can be easily recoverable using an external magnetic field, these promising potentials have rendered any successful effort to improve their photocatalytic efficiency in substantiating their practical system uses [29].

It was previously noted that the structural features and magnetic, electrical characteristics and catalytic activity of ferrites could be dependent on the metals in the ferrite lattice structure [30]. In addition, several studies have reported the enhancement in the catalytic activity of ferrites with different metal substitution and change in cation distribution. For instance, the photocatalytic activity of cobalt zinc ferrite systems on Mn substitution has been documented by Santosh Bhukal et al. [31], showing that the decolorization ratio of methyl orange is enhanced along with increasing Mn^3+^ ions content. In another study, MgFe_2_O_4_ doped with Co^2+^ ions by modified sol-gel combustion method exhibited higher degradation efficiency for methylene blue in comparison with that of pure MgFe_2_O_4_ sample [32]. Similar results were obtained with nickel ferrite when substituted with Zn [33].

On the other hand, the substitution that gained attention in doping ferrite iron was rare-earth ions [34,35,36,37,38]. Since these metals have a strong spin-orbit coupling of angular momentum due to the presence of unpaired electrons in the 4f orbitals, their interaction with ferrites occurs in 3d-4f coupling, resulting in magneto-crystalline anisotropy and thus influencing magnetic, electrical and catalytic features of substituted ferrites [39]. Mariosi et al. found that cobalt ferrite nanoparticles substituted by La^3+^ ions exhibited structural changes in terms of cationic arrangement of the spinel structure [38]. This change resulted in a decrease in coercivity values and an increase in the surface area. The substitution of other rare-earth ions such as La^3+^, Nd^3+^, Gd^3+^ and Dy^3+^ into the [B] sites containing iron has been shown to displace Fe^3+^ into (A) sites, thus altering the structure and electrical and magnetic characteristics the ferrites [34,35,37,39]. Sharma et al. have carried out one prominent study showing the ability of rare-earth doping to boost catalytic activity [40]. Specifically, rare-earth (La^3+^, Ce^3+^) substituted CoFe_2_O_4_ exhibited higher efficiencies in the elimination of five model pollutants, possibly due to the presence of Ce^3+^/Ce^4+^ redox pair. The synthesis of samarium (Sm^3+^) substituted manganese ferrite nanoparticles (MnFe_2_-_x_Sm_x_O_4_) using oleic acid as a surfactant was reported by Rashmi et al. [41]. Such synthesized nanomaterials were tested for photocatalytic degradation of colors under visible light irradiation. Such synthesized nanomaterials were tested for photocatalytic degradation of colors under visible light irradiation. The result indicated that samarium replacement significantly increased the photocatalytic activity of MnFe_2_O_4_ nanoparticles. The value of x varied from 0, 0.5, 1.0, 1.5 and 2.0, and the best results were obtained at x = 1.5. The higher activity of x = 1.5 was related to its minimum band gap energy value (1.64 eV). After that, Patil et al. [42] synthesized Gd^3+^ doped ZnFe_2_O_4_ (ZnFe_2−x_Gd_x_O_4_) nanoparticles via coprecipitation method. Synthesized photocatalysts were checked for MB photo-degradation, resulting in enhanced degradation of MB, from about 95 to 99% in the presence of photocatalysts ZnFe_2−x_Gd_x_O_4_ (x = 0, 0.3, 0.5 and 0.7) along with 8 ppm of H_2_O_2_. The efficiency level was found higher than that of the pristine ZnFe_2_O_4_ and could be attributable to the fact that Fermi energy levels of substituted catalyst were just below the conduction band within the energy band gap. In addition, formation of lattice strains due to the difference between ionic radii of Gd^3+^ (0.94 Å) and that of Fe^3+^ (0.78 Å) is also partially responsible for the enhancement. However, the impact of rare earth substitution (e.g., Nd) on the photo-Fenton activity of ferrites for dye degradation is still a gap in the literature.

The present study aims to investigate the structural and catalytic properties of ferrites by the doping substitution of Nd^3+^ ions. ZnFe_2_O_4_ was incorporated with various Nd^3+^ molar ratio (0–0.05 mol%) using urea as a fuel additive. The as-synthesized ferrite was then characterized using several techniques (XRD, SEM, TEM, EDX, and FT-IR before being tested for photocatalytic activities toward Rhodamine B. The effect of catalyst loading, H_2_O_2_ concentration, and contact time on the photo catalytic activity of ZnFe_2_O_4_ nanoparticles was surveyed meticulously.

## 2. Materials and Methods

### 2.1. Synthesis of ZnNd_x_Fe_2−x_O_4_ Nanoparticles

Firstly, urea coprecipitation method was adopted to fabricate nanocrystalline ZnNd_x_Fe_2−x_O_4_ (x = 0, 0.01, 0.03, 0.05) ferrites according to a previous publication with a moderate modification [34]. Analytical grade zinc nitrate tetrahydrate [Zn(NO_3_)_2_·4H_2_O, 98% pure, Sigma-Aldrich, Darmstadt, Germany], iron(II) nitrate nonahydrate [Fe(NO_3_)_3_·9H_2_O, 99.9% pure, Sigma-Aldrich], and neodymium(III) nitrate hexahydrate (Nd(NO_3_)_3_·6H_2_O, 99.9% pure, Sigma-Aldrich) were used as oxidizer and urea (CH_4_N_2_O, >99% pure, Sigma-Aldrich) was used as a fuel additive. A number of proper ions: Zn^2+^ (1 mmol) combined with Fe^3+^ [(2−x) mol] and Nd^3+^ (x mol) (x = 0, 0.01, 0.03, 0.05) were dissolved in distilled water. The final pH of the solution was adjusted at 5.0, and then was heated to 100 °C for 1 h. The precipitation was collected, washed with distilled water (3 × 20 mL) to eliminate metal ion and anion traces and then calcinated up to 500 °C for 2 h (ramping rate of 10 °C/min). The product was ground and stored at a vacuum container.

### 2.2. Characterization

The as-synthesized particles were characterized using a number of techniques including X-ray diffraction (XRD), Fourier-transform infrared spectroscopy (FT-IR), scanning electron microscope (SEM), transmission electron microscopy (TEM), energy dispersive X-ray spectroscopy (EDX) and UV-Vis absorption spectroscopy. Respective instruments for those analyses include D8 Advance diffractometer (Brucker, Madison, WI, USA) with CuK_α_ radiation (*λ* = 1.5406 Å) in a 2θ angle ranging from 20° to 70° with a step of 0.03° source, FTIR Affinity-1S (Shimadzu, Kyoto, Japan), Hitachi S-4800 (Tokyo, Japan), JEOL-JEM-1010 (Tokyo, Japan), JEOL JED 2300 Analysis Station (Tokyo, Japan) and U-4100 (Hitachi, Tokyo, Japan) operating in the wavelength range of 200–800 nm.

### 2.3. Photocatalytic Degradation of Rhodamine B

In this study, Rhodamine B (RhB) was used as target pollutant to assess the photocatalytic potential of as-synthesized ferrite samples. Accordingly, the RhB degradation reaction was carried out in a reactor containing the ZnNd_x_Fe_2−x_O_4_ (x = 0, 0.01, 0.03, 0.05) nanoparticles and RhB dye under visible light irradiation (using 30 W Led lamps, Philips, Amsterdam, Netherlands). In a typical experiment, 0.1 g of catalyst was introduced into 200 mL of RhB aqueous solution (10 mg·L^−1^) and suspended on a shaker table at 200 rpm. The suspension was first stirred in the dark for 30 min to attain the adsorption-desorption equilibrium state between the catalyst and RhB. Afterwards, the reaction was stirred and H_2_O_2_ 30% (*w*/*w*) in H_2_O (Sigma-Aldrich) was added to the mixture, which was then irradiated under visible light for 210 min. To determine the RhB concentration in the mixture, 5 mL of each aliquot was taken out periodically, then centrifuged to remove the solid catalyst. The effect of two factors including H_2_O_2_ concentrations (0.02 M, 0.04 M and 0.06 M) and the catalyst dosages (0.5, 0.75 and 1.0 g/L) on the photo-degradation efficiency was studied.

The efficient degradation of RhB (H) was calculated according to the formula Equation (1).

(1) $$H=\frac{C_{o}-C_{t}}{C_{o}}\times 100$$

where C_o_ and C_t_ are the concentration of RhB (mg·L^−1^) at the time 0 and t. The samples were measured by scanning at the maximum wavelength λ = 553 nm.

## 3. Results

### 3.1. Characterization

**Figure 1:**
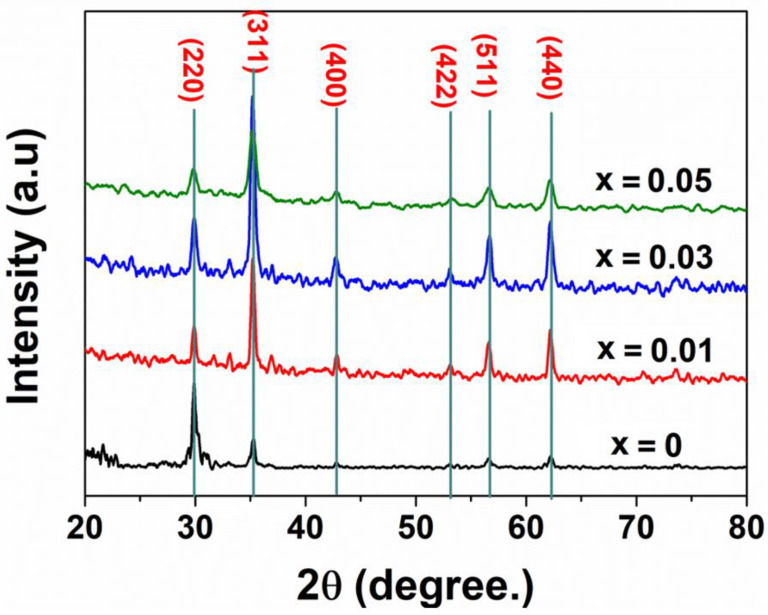
X-ray diffraction patterns of ZnNd_x_Fe_2−x_O_4_ nanoparticles (x = 0.0–0.05) annealed at 500 °C.

Figure 1 illustrates XRD patterns of ZnFe_2_O_4_ and different ZnNd_x_Fe_2−x_O_4_ (x = 0.01, 0.03, 0.05) samples synthesized at 500 °C. The formation of zinc ferrite (JCPDS number 022-1012) was evidenced by reflection peaks corresponding to the characteristic spacing between (220), (311), (400), (422), (511) and (440) planes of a cubic spinel structure. Employing Scherrer’s equation, average crystallite size of the samples could be calculated as follows:

(2) $$D_{\mathrm{XRD}}=\frac{k\lambda }{\beta \cos\theta }$$

where *λ*, *k*, *β* and *θ* wavelength of the X-ray (0.1504 nm), the Scherrer’s constant (*k* = 0.89), the full width at half maximum observed in radians and the angle of diffraction of the (311) peak with the highest intensity, respectively.

To determine the lattice constant (a) at the most intense peak (311), following formula was used

(3) $$a=d_{\mathrm{hkl}}\sqrt{h^{2}+k^{2}+l^{2}}$$

where d is interplanar distance and h, k, l are Miller indices. The change in crystalline structure with doping of Nd^3+^ ions could be observed from the XRD data given in Table 1. It clearly indicates that the average crystallite sizes significantly decrease from 22 mm to 12 nm with increasing the content of Nd^3+^ from 0 to 0.05 mol, which was in good agreement with a previous publication [36]. Moreover, the lattice constants for the samples of zinc ferrites nanoparticles increase slightly from 8.43 to 8.45 Å as the amount of Nd^3+^ added increases. This outcome can be due to the difference of the radius of ferrites, or more specifically, metal ions radius (Nd^3+^, Zn^2+^, Fe^3+^). Indeed, the ionic radius of Nd^3+^ ion (0.98 Å) is larger than the ionic radius of Zn^2+^ (0.74 Å) and Fe^3+^ (0.67 Å); hence Nd^3+^ ions prefer to occupy more octahedral sites (B-sites) than Fe^3+^ ions [36]. It is likely for Nd^3+^ ions to be distributed in the grain boundaries, thus contributing to the improvement of energy barrier of Zn^2+^ or Fe^3+^ movement [38]. As a result, the growth of ferrites nanoparticles grains and the crystallite size of zinc ferrites tends to decrease while their crystal lattice constant increases. The same phenomena in decreasing crystallite size due to increasing rare-earth ions content have been observed previously in cobalt ferrites [38,39], nickel ferrites [37] and zinc ferrites [36]. To sum up, the dope of Nd^3+^ showed a significant effect on the crystalline nanostructure of origin zinc ferrites.

**Figure 2:**
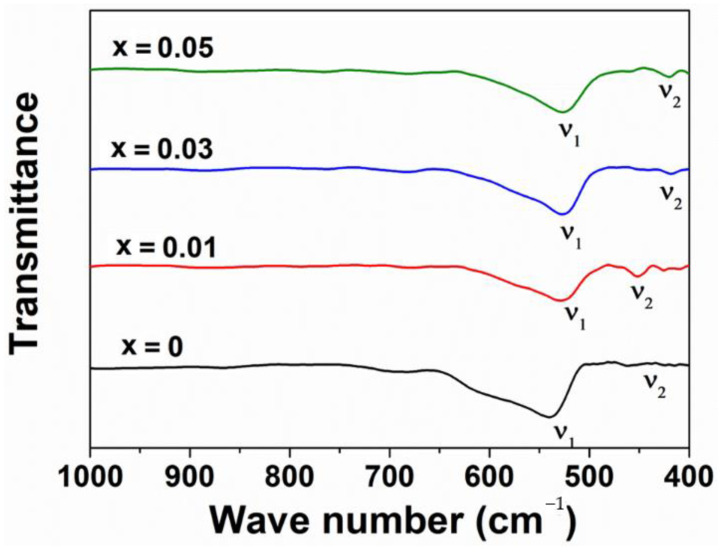
FT−IR spectrum of ZnNd_x_Fe_2−x_O_4_ nanoparticles.

Chemical bonds diagnosed from the FT-IR spectra in Figure 2 can suggest two most characteristic bands for the as-synthesized ZnNd_x_Fe_2−x_O_4_ nanoparticles. The first band is located at 522.7−528.6 cm^−1^ (Table 1), which corresponds to the stretching vibration in the tetrahedral bonding of Zn-O [26,37]. The other band appeared at 418.5–451.3 cm^−1^, which is attributable to the stretching frequency of the octahedral bonding of Fe−O and Nd−O. The change in the lattice parameters can reflect the shift of the band vibrations. The position and intensity of ν_1_ and ν_2_ tend to change with increasing Nd^3+^ ions content. Finally, the frequency change confirms the presence of the Nd^3+^ ions occupying the octahedral sites in the ferrites lattice.

**Figure 3:**
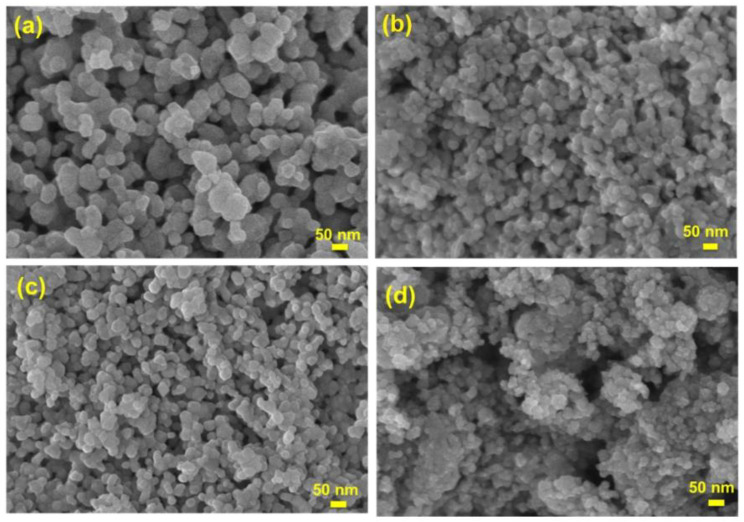
SEM photomicrography of ZnNd_x_Fe_2−x_O_4_ nanoparticles: (**a**) x = 0, (**b**) x = 0.01, (**c**) x = 0.03, (**d**) x = 0.05.

To better understand the structure of samples, the morphology of ZnNd_x_Fe_2−x_O_4_ nanoparticles is observed by SEM technique. The samples including pure ZnFe_2_O_4_ and synthesized ZnNd_x_Fe_2−x_O_4_ nanoparticles (x = 0.01; 0.03 and 0.05) all display a type of uniform sphere (Figure 3). The crystallite size of the zinc ferrites decreases with increasing Nd content, which is consistent with the result of XRD analysis.

**Figure 4:**
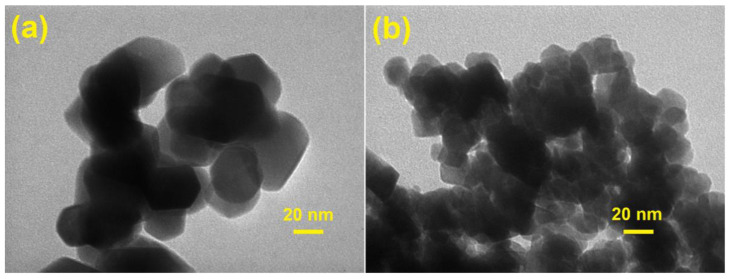
TEM of ZnNd_x_Fe_2−x_O_4_ nanoparticles: (**a**) x = 0; (**b**) x = 0.03.

**Figure 5:**
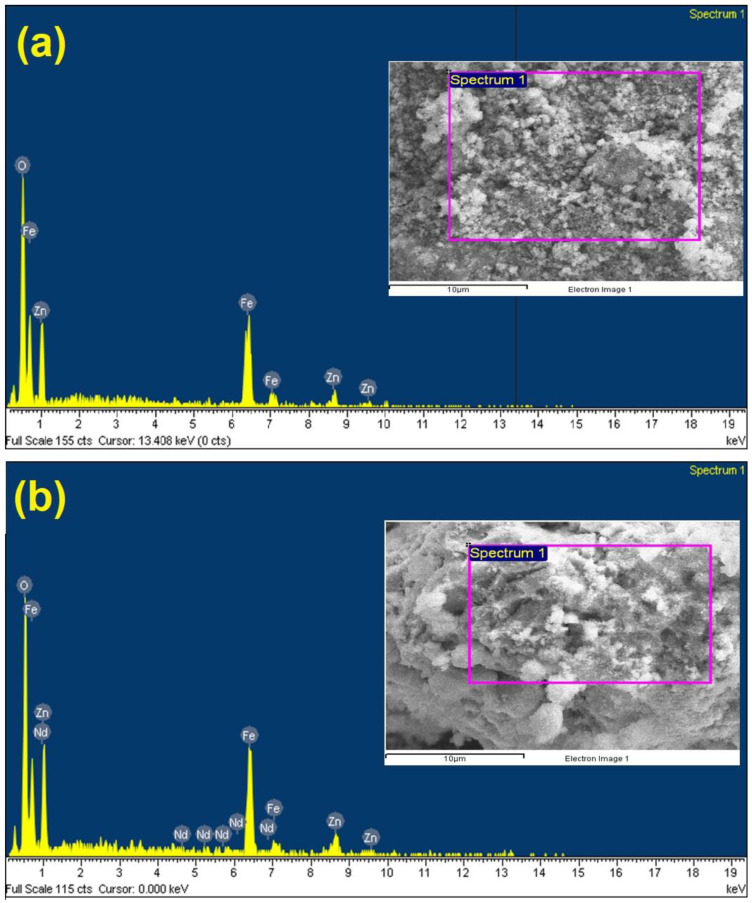
EDX spectra of ZnNd_x_Fe_2−x_O_4_ nanoparticles: (**a**) x = 0; (**b**) x = 0.03.

Figure 4 displays the TEM photomicrography of the pure ZnFe_2_O_4_ and ZnNd_0.03_Fe_1.97_O_4_ annealed at 500 °C. Both samples ZnNd_0.03_Fe_1.97_O_4_ and ZnFe_2_O_4_ exhibit mostly homogeneous microspheres. In particular, the agglomeration or clustering of these microspheres is rarely observed. Although the effect of Nd^3+^ ions on the morphology is almost inconsiderable, the particle size of ZnNd_0.03_Fe_1.97_O_4_ sample is smaller than that of the ZnFe_2_O_4_ sample. The grain size from TEM studied is the close agreement with the XRD data and SEM photomicrography. Moreover, the chemical composition of samples was confirmed by EDX spectra. The presence of all elements in the XRD profile indicates that synthesized material was of high purity (Figure 5).

**Figure 6:**
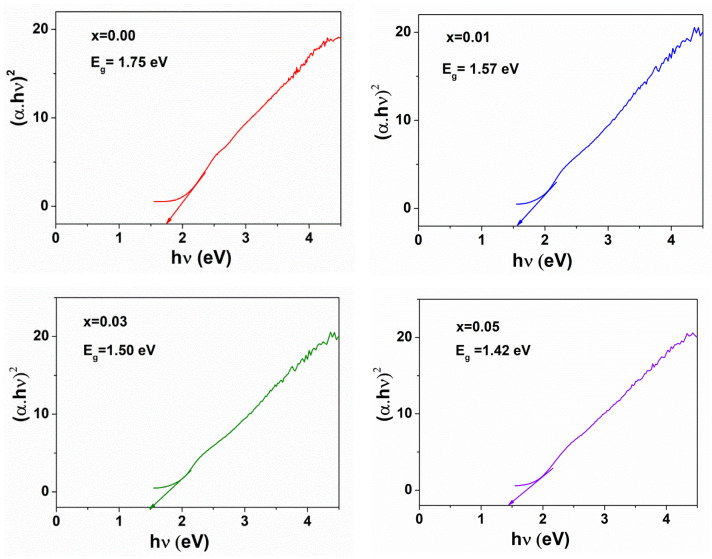
The band gap energies of ZnNd_x_Fe_2−x_O_4_ nanoparticles (x = 0.00–0.05).

The band gap of the spinel nanoparticles was determined by DRS. Kubelka-Munk model was used to calculate band gaps (E_g_) of zinc ferrites nanoparticles with the absorption coefficient (α) obtainable from DRS spectra as Equation (4).

(4) $$F(R)=\alpha =\frac{{\left(1-R\right)}^{2}}{2R}$$

where, F(R) represents the Kubelka-Munk function, *α* is the absorption coefficient and R is the reflectance. The following relationship could be used to determine the band gap energy (E_g_) as shown in Equation (5).

(5) $$\alpha h\nu =A{\left(h\nu -E_{g}\right)}^{n}$$

where, h*ν*: energy of the photon, *α*: the the absorption coefficient, A: material parameter and n: transition parameter, n = 2 represent indirect transitions. The slope of plotting (αh*ν*)^2^ against h*ν* could be used to measure the band gap energy for the absorption peak, as shown in Figure 6. The band gap values of ZnNd_x_Fe_2−x_O_4_ (x = 0, 0.01, 0.03, 0.05) nanoparticles are found to be 1.75, 1.57, 1.50 and 1.42 eV, respectively. This indicates that the Nd^3+^ ions concentration affected the band gap energy of zinc ferrites nanoparticles. The band gap energy value decreased with increasing the Nd^3+^ ions concentration. Due to the larger ionic radius, the crystal lattice is bound to distort leading to generation of interface defects [38]. In zinc ferrites nanoparticles, the orbital overlapping between O-2p and Fe-3d energy levels caused the formation of the energy band gap. There is the 4f Fermi energy level of Nd in ZnNd_x_Fe_2−x_O_4_ samples, thus resulting in decreased band gap energy value [42,43]. The optical band gap of the CoFe_2_O_4_ samples doped with La decreases from 1.35 to 1.1 eV [38].

### 3.2. Photocatalytic Activity

#### 3.2.1. Influence of Experimental Conditions

**Figure 7:**
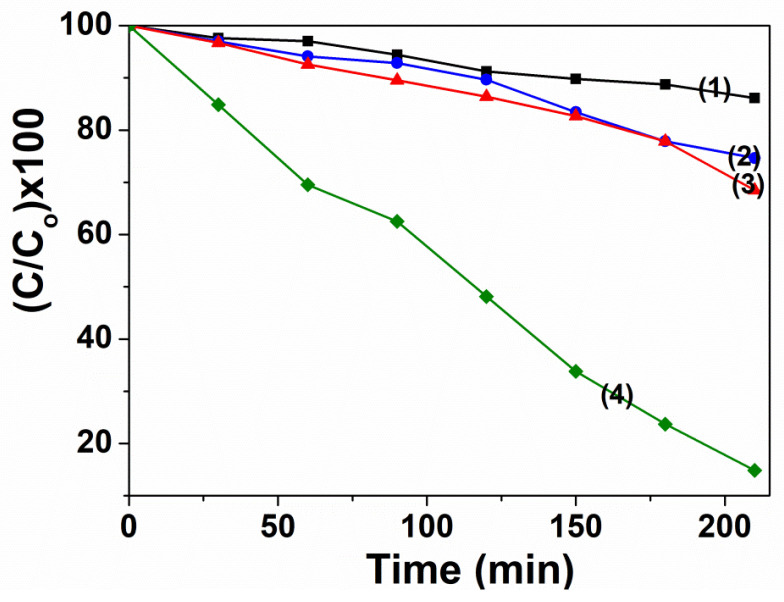
The photocatalytic degradation of RhB in different conditions: curve (1), 10.0 mg/L RhB + 0.04 M H_2_O_2_ + light; curve (2), 10.0 mg/L RhB + 0.1 g ZnFe_2_O_4_ + light; curve (3), 10.0 mg/L RhB + 0.1 g ZnFe_2_O_4_+ 0.04 M H_2_O_2_ + dark; curve (4), 10.0 mg/L RhB + 0.1 g ZnFe_2_O_4_ + 0.04 M H_2_O_2_ + light.

The photo-Fenton catalytic degradation activities of ZnFe_2_O_4_ catalyst occurring at different experimental parameters are illustrated in Figure 7. The lowest RhB removal efficiency is 13.87%, reached only when there is only H_2_O_2_ existed in the solution. Under ZnFe_2_O_4_/Visible-light system, the decolorization ratio achieved 25.35%. This figure was enhanced to 31.51% when ZnFe_2_O_4_ combined with H_2_O_2_. However, the removal rate of RhB reaches to 85.14% under irradiation and in the presence of ZnFe_2_O_2_ and H_2_O_2_. The high removal rate could be explained by the h^+^ in the valence of ZnFe_2_O_4_ and photodecomposition of H_2_O_2_ that produce more •OH. Both of which contributed to the improved oxidation of dyes [22]. On the other hand, the production of •OH could also be promoted by decreased recombination of electrons and holes, caused by the participation of photo-induced electrons in the Fe^3+^/Fe^2+^ cycle reaction [26].

**Figure 8:**
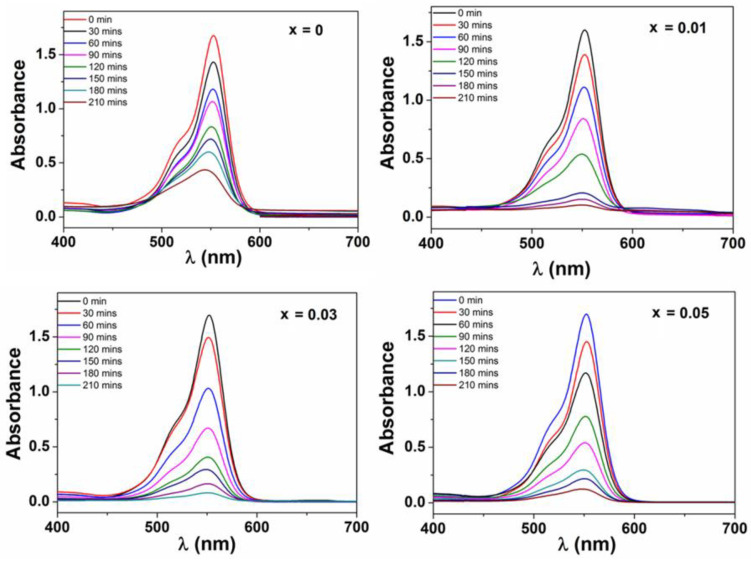
The change in absorption of Rhodamine B solution with time in the presence of H_2_O_2_ and ZnNd_x_Fe_2−x_O_4_ nanoparticles (x = 0–0.05) under irradiation.

**Figure 9:**
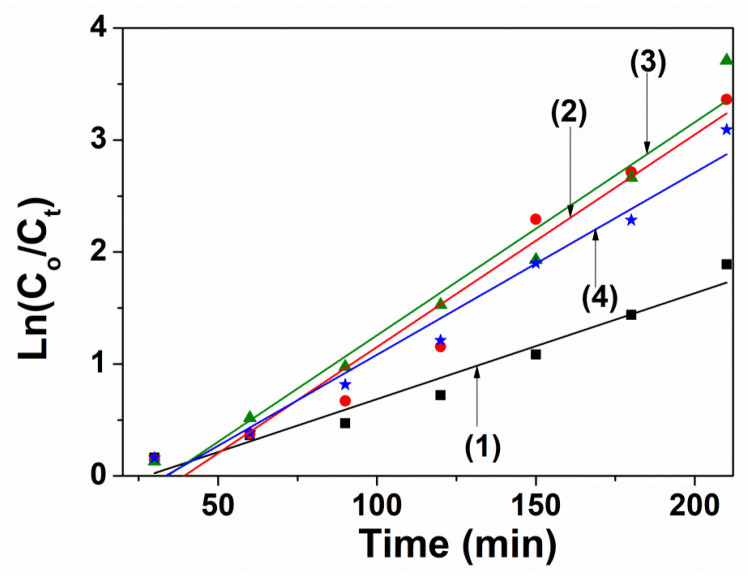
The plots of ln(*C_o_*/*C_t_*) versus irradiation time (*t*) in the presence of H_2_O_2_ and ZnNd_x_Fe_2−x_O_4_ nanoparticles: (1) x = 0, (2) x = 0.01, (3) x = 0.03, (4) x = 0.05.

When the ZnFe_2_O_4_ crytals are doped with Nd^3+^ ions, their photocatalytic degradation of RhB are enhanced. The higher photocatalytic performance at higher Nd^3+^ introduced may be due to smaller crystallite sizes from 22 to 12 nm with increasing Nd^3+^ ions content. This may be leading to the larger surface area and higher amount of active photocatalytic sites. Moreover, another effect is band gap energy value decreasing from 1.75 to 1.42 eV with increasing Nd^3+^ ions concentration, which aids the formation of ∙OH active species and stimulates oxidative degradation of dye molecules. UV–vis absorption spectra of RhB during the degradation by ZnNd_x_Fe_2−x_O_4_ (x = 0–0.05) at the different irradiation time as shown in Figure 8. The photocatalytic degradation efficiency of RhB and kinetic constant after 210 min irradiation are 96.53% and 0.0095 min^−1^, 98.00% and 0.0189 min^−1^, 95.46% and 0.0163 min^−1^ in the presence of H_2_O_2_ and ZnNd_x_Fe_2−x_O_4_ with x = 0.01, 0.03 and 0.05, respectively (Figure 9 and Table 2). This phenomenon can rely on the combination of rare earth ions and the ions in the crystal lattice of ferrite to generate the energy levels and the defects, which has been confirmed by XRD and DRS measurements [38,39].

#### 3.2.2. Influence of H_2_O_2_ Concentration

**Figure 10:**
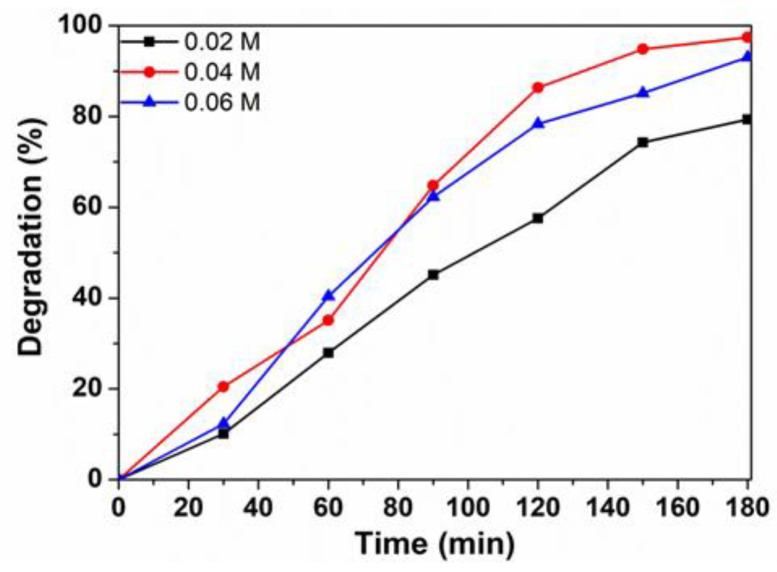
The influence of H_2_O_2_ concentration on the efficient degradation of Rhodamine B using ZnNd_0.03_Fe_1.97_O_4_ as Photo-Fenton.

Figure 10 shows the removal efficiency of RhB under different concentrations of H_2_O_2_. When initial H_2_O_2_ concentration increased from 0.02 M to 0.04 M, the degradation efficiency increased from 79.4% to 97.42%. However, the degradation efficiency decreased to 93.02% when H_2_O_2_ concentration increased to 0.06 M. The initial increase in the degradation could be explained due to the generation of the higher number of ^•^OH active species which are mainly responsible for the oxidative degradation of dye molecules, Equation (3). When hydrogen peroxide presents in high concentration, ^•^OH could be scavenged (Equations (4)–(6)) and reduced [22,26]. Therefore, the degradation efficiency of RhB dye is greatly reduced. The optimal initial H_2_O_2_ content was 0.04 M.

H_2_O_2_ → 2^•^OH (6)

^•^OH + H_2_O_2_ → ^•^OOH + H_2_O (7)

^•^OH + ^•^OH → H_2_O_2_ (8)

^•^OH + ^•^OOH → H_2_O + O_2_ (9)

#### 3.2.3. Influence of the Catalyst Loading

**Figure 11:**
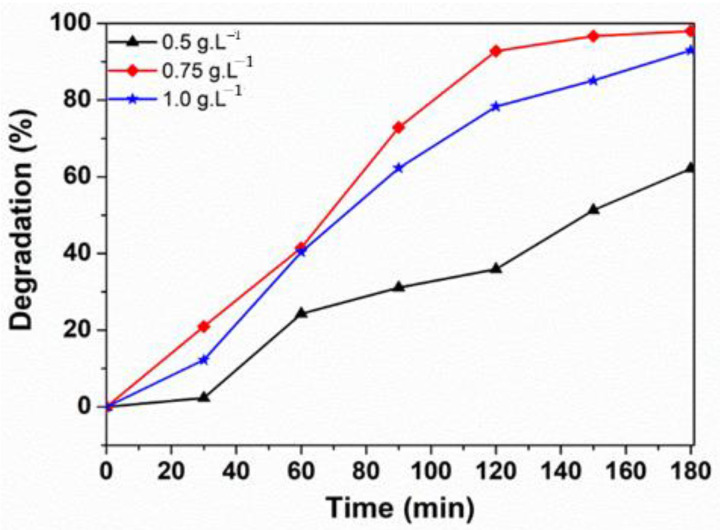
The effect of ZnNd_0.03_Fe_1.97_O_4_ dosage on the degradation of Rhodamine.

The effect of the ferrite sample amount on the RhB removal rate is shown in Figure 11. When the ZnNd_0.03_Fe_1.97_O_4_ dosage increases from 0.5 g/L to 0.75 g/L, the efficient degradation of RhB increases from 62.13% to 98.01% at 180 min. However, the removal rate of RhB decreased to 93.02% when increasing the ZnNd_0.03_Fe_1.97_O_4_ dosage to 1.0 g/L. This outcome is because when increasing catalyst dosage, ^•^OH radical amount increases due to the reaction of h^+^ in the valence of ferrite sample [22]. However, with a high catalyst dosage, the degradation efficiency of RhB dye decreases due to increased solution turbidity, in turn obstructing light irradiation and activating the totality of the catalyst suspension [43]. Therefore, the optimal catalyst dosage was 0.75 g/L.

## 4. Conclusions

Nd^3+^ substituted zinc ferrite nanoparticles were successfully synthesized via solution combustion technique. The physical and chemical characteristic of the ZnNd_x_Fe_2−x_O_4_ samples were investigated by XRD, EDX, FT-IR, SEM and TEM. The average crystallite size and the optical band gap values reduced from 22 to 12 nm and from 1.75 to 1.42 eV, respectively, with increasing Nd^3+^ ions content. The substitution of Nd^3+^ ions on octahedral sites was confirmed by the change of ν_1_ and ν_2_ frequency. The enhanced photocatalytic activity of the zinc ferrite samples was observed with increasing Nd^3+^ ions concentration. The ZnNd_0.03_Fe_1.97_O_4_ nanoparticles have the highest efficient degradation for Rhodamine B. The removal efficiency of Rhodamine B dye was affected by the concentration of H_2_O_2_, catalyst amount. The optimal initial H_2_O_2_ content was 0.04 M and the optimal dosage of the catalyst was 0.75 g/L.

## Figures and Tables

**Table 1:** Average crystallite size (D_XRD_), lattice parameter (a), unit cell volume (V) and wave number, ν_1_ and ν_2_ for the tetrahedral and octahedral of the ZnNd_x_Fe_2−x_O_4_ samples, respectively.

Samples	D_XRD_ (nm)	a (Å)	V (Å^3^)	ν_1_ (cm^−1^)	ν_2_ (cm^−1^)
ZnFe_2_O_4_	22	8.43	599.08	522.7	447.5
ZnNd_0.01_Fe_1.99_O_4_	21	8.44	601.21	528.5	451.3
ZnNd_0.03_Fe_1.97_O_4_	18	8.45	603.35	526.0	418.5
ZnNd_0.05_Fe_1.95_O_4_	12	8.45	603.35	526.6	420.5

**Table 2:** The degradation efficiency (H%) and pseudo first-order rate constant (k) for the photocatalytic degradation of RhB in the presence of H_2_O_2_ 0.04 M using ZnNd_x_Fe_2−x_O_4_ nanoparticles.

Samples	H (%)	k (min^−1^)	R^2^
ZnFe_2_O_4_	85.14 ± 0.99	0.0095	0.952
ZnNd_0.01_Fe_1.99_O_4_	96.53 ± 0.95	0.0189	0.951
ZnNd_0.03_Fe_1.97_O_4_	98.00 ± 0.44	0.0190	0.964
ZnNd_0.05_Fe_1.95_O_4_	95.46 ± 0.91	0.0163	0.972

## Data Availability

The data presented in this study are available on request from the corresponding author.
